# The Role of Initial Clinical and Laboratory Findingsin Infants With Hyperthyrotropinemia to PredictTransient or Permanent Hypothyroidism

**DOI:** 10.4274/Jcrpe.931

**Published:** 2013-09-18

**Authors:** Tolga Ünüvar, Korcan Demir, Ayhan Abacı, Atilla Büyükgebiz, Ece Böber

**Affiliations:** 1 Dokuz Eylül University Medical Faculty, Department of Pediatric Endocrinology, İzmir, Turkey; 2 Bilim University, Department of Pediatric Endocrinology, İstanbul, Turkey

**Keywords:** Permanent congenital hypothyroidism, hyperthyrotropinemia, thyroxine, infant

## Abstract

**Objective:** Studies on the clinical course of children with hyperthyrotropinemia are scarce. We aimed to evaluate the role of presentation findings in such infants to predict eventual outcome.

**Methods:** Files of infants diagnosed as suspicious congenital hypothyroidism (CH) in the neonatal or early infancy period in the past ten years were analyzed retrospectively, and 37 patients (M/F: 20/17) with hyperthyrotropinemia diagnosed at a median age of 3.2 months were included in the study. Criteria for inclusion were: normal free thyroxine (fT4) levels and thyrotropin (TSH) levels between 10-20 μIU/mL during the initial neonatal screening (or TSH<10μIU/mL afterwards). Cases with permanent CH (Group 1) were compared to those with transient hyperthyrotropinemia (Group 2) regarding age at the time of diagnosis, sex, gestational age, birth weight, symptoms, ultrasonographic and scintigraphic findings, initial thyroid function tests, and state of mental and motor development.

**Results:** Of the total group, 20 patients (54%) were eventually diagnosed as permanent CH. T4 doses that maintained normal thyroid function tests were significantly higher at the end of the first and second years of life in this group. Age, TSH and fT4 levels at the time of diagnosis, sex, gestational age, birth weight, symptoms, ultrasonographic and scintigraphic findings, and the state of mental and motor development were similar in the two groups.

**Conclusions:** T4 dose required to maintain a euthyroid state was the only parameter which distinguished between transient and permanent CH.

**Conflict of interest:**None declared.

## INTRODUCTION

Owing to screening programs which have been applied in developed countries in the past 30 years, congenital hypothyroidism (CH) is diagnosed and treated at an early age and no longer presents a social problem in these countries. However, CH is still a huge public health problem and an important cause of mental retardation in iodine-deficiency regions and in underdeveloped countries in which screening programs cannot be applied (1,2,3).

Transient hypothyroidism has become a commonly encountered condition in parts of the world where newborn screening tests are a routine procedure for all infants. Studies from Japan and Europe report that the incidence of hyperthyrotropinemia is 1/17000 and 1/8260, respectively (4,5). Approximately 10 percent of the infants with a diagnosis of CH are subsequently identified as cases of “transient CH” (6). The pathogenesis of neonatal transient hypothyroidism is multifactorial. Several genetic and environmental conditions including extrauterine adaptation stress on an immature pituitary-thyroid axis due to prematurity, perinatal goitrogen exposure, intrauterine maternal anti-thyroid drug exposure, maternal thyrotropin (TSH) receptor blocker antibodies, heterozygote thyroid oxidase-2 deficiency, TSH receptor mutations, endemic iodine deficiency or perinatal iodine over-exposure can all lead to this condition (1,2,3,4,5,6,7). Additionally, isolated hyperthyrotropinemia [normal free thyroxine (fT4) levels and elevated TSH] may be observed in infants whose blood was obtained in the first 1-2 days after birth (physiologic TSH increase) (1,8). If the TSH increase is continuous, the infant is diagnosed to have “persistent hyperthyrotropinemia”. There exists no method to predict the outcomes of infants with hyperthyrotropinemia (9,10).

In the medical literature, there is a lack of detailed studies on the clinical course of infants with hyperthyrotropinemia. Recommendations regarding management of hyperthyrotropinemia are as follows: (i) to recheck TSH level as soon as possible, (ii) to investigate for factors that might cause hyperthyrotropinemia, and (iii) to start treatment if TSH remains elevated. The guidelines of the American Academy of Pediatrics on newborn screening and treatment of CH define all newborns presenting with TSH serum levels persistently higher than 10 mU/L as affected by CH and requiring treatment (1). Niwa et al (11) have shown that hyperthyrotropinemia (TSH>10 mU/L) is a marker of thyroid dysfunction in premature infants aged two weeks or older. However, there is no consensus on treatment of persistent hyperthyrotropinemia with TSH levels between 5 and 10 mU/L.

Here, we aimed to evaluate (i) the role of presentation findings in such infants in predicting eventual outcome and (ii) the clinical and hormonal characteristics that may help to distinguish between CH and hyperthyrotropinemia in the neonatal and infancy periods.

## METHODS

Files of a sample of infants diagnosed as suspicious CH during neonatal or early infancy period between 1997 and 2007 were analyzed retrospectively, and infants with TSH levels between 10 μIU/mL and 20μIU/mL were identified. A total of 37 patients (M/F: 20/17) with hyperthyrotropinemia diagnosed at the median age of 3.2 months (25th-75th p; 0.9-6 months) were included in the study. These infants had normal fT4 levels and TSH levels between 10 μIU/mL and 20 μIU/mL during the first three weeks of life (or TSH levels between 5 μIU/mL and 10 μIU/mL afterwards). Patients with Down’s syndrome and secondary or tertiary hypothyroidism were excluded from the study. Clinical and laboratory data during follow-up were recorded. In routine follow-up, treatment is discontinued at age 2-3 years in all patients with mild thyroid dysfunction and the patient is reevaluated. Patients with normal thyroid function tests after discontinuation of treatment for one month following 2-3 years of regular treatment, and patients in whom treatment needed to be stopped due to biochemical hyperthyroidism were diagnosed as cases of hyperthyrotropinemia. Cases found to have permanent CH (Group 1) were compared to those with hyperthyrotropinemia (Group 2) regarding age at diagnosis, sex, gestational age, birth weight, symptoms, ultrasonographic and scintigraphic findings, initial thyroid function tests, and mental/motor development. The results of the WISC-R and Denver mental tests were evaluated by a pediatric psychiatrist. Elecsys reagent kits were used to measure the TSH, fT4, free triiodothyronine (T3), total T4, and total T3 levels. They were analyzed by Electrochemiluminescence Immunoassay “ECLIA” method and Roche Elecsys E170 device.TSH levels between 0.4-5 µIU/mL and fT4 levels between 0.8-1.9 ng/dL were accepted as “normal”.

**Statistical Analysis**

The statistical analysis was performed by SPSS (Statistical Package for the Social Sciences) 11.5 package program. First, descriptive analyses were performed. The variables without normal distribution were presented as median (25%-75%), and the variables with normal distribution were presented as mean±standard deviation. Chi-square test, Student’s t-test (if the data distribution is normal) and Mann-Whitney U-test (if the data distribution is not normal) were used to compare the groups. A p-value of <0.05 was chosen to represent statistical significance.

## RESULTS

There was no family history of thyroid disease and associated malformations in any of the patients. Twenty (54%) of the patients were eventually diagnosed as permanent CH. Age, TSH and fT4 levels at the time of diagnosis, sex, gestational age, birth weight, symptoms, ultrasonographic and scintigraphic findings, and level of mental/motor development at the end of follow-up were similar in the two groups. Thyroid ultrasonography and Tc99m scintigraphy were evaluated as abnormal (thyroid hypoplasia) in 4 patients in Group 1. Ultrasonography and scintigraphy findings were normal in all patients in Group 2. The difference between the two groups was not of statistical significance. T4 doses (μg/kg/d) that maintained euthyroidism were significantly higher at the end of first and second years in Group 1 (Table 1). Prolonged jaundice was the most frequent presenting complaint in both groups (p=0.373). Follow-up and treatment could be carried out after age two years in 11of the 20 patients (68.8%) in Group1, this rate was insignificantly lower in Group 2 (n=5, 31.3%) (p=0.375). Mean treatment period was 30 months in subjects with hyperthyrotropinemia. Hyperthyoridism due to overtreatment was detected in 10 of the patients with hyperthyrotropinemia, while this was not observed in any of the patients with permanent CH. As expected, TSH levels after one month of discontinuation of L-T4 were significantly higher in Group 1.

## DISCUSSION

The early diagnosis and treatment of CH is of utmost importance. Delayed diagnosis and treatment may lead to neurological sequelae including mental retardation, poor motor coordination, ataxia, spastic diplegia, muscular hypotonia, strabismus, learning difficulties and attention deficit disorders (1,2,3). With increased application of screening for CH in the newborn in many countries including Turkey, transient hypothyroidism has become a commonly encountered condition. Hashemipour et al (12), in a study conducted in Iran, reported the incidence for transient CH as 40.2% of all cases with high TSH levels. In a study conducted in Turkey, 30 percent of 182 newborns with high TSH levels were found to have transient CH (13). Parks et al (14) stated that the recent reported increase in the incidence of CH in the United States was due to the erroneous evaluation of cases with transient hypothyroidism as “persistent”. This false evaluation has also led to unnecessary administration of long-term L-T4 therapy. Therefore, it is very important to make a rapid and accurate differential diagnosis in newborns with high TSH levels.

Studies on the management of transient hypothyroidism are limited. This patient group was misdiagnosed as persistent CH in the early period and received T4 treatment unnecessarily. The present study aimed to investigate whether there exist any differences in clinical or laboratory findings to differentiate these two groups in the early period. In a study conducted by Zung et al (4), mean weight and gestational ages were significantly lower in patients with hyperthyrotropinemia as compared to patients with CH. In our study, no significant differences in these parameters were found between the two groups. Therefore, we believe that it is important to remember the possibility of transient hyperthyrotropinemia in mature, non-SGA healthy neonates as well. Zung et al (4) also reported significant differences in fT4 levels, thyroid ultrasonography and scintigraphy findings between preterm and term newborns. In our study, these parameters were similar in the groups. Yang et al (15) reported that physical and mental growth following treatment discontinuation in patients with transient hypothyroidism (when they were 2-3 years of age; at the 1-year follow-up) were similar to that of healthy children. Similarly, Demirel et al (16) found age-appropriate normal results in the Denver developmental screening test in patients with hyperthyrotropinemia. Our results show no significant differences in the Denver and WISC-R tests between the patients with persistent CH and those with transient hyperthyrotropinemia. This finding also indicates that optimal treatment and close follow-up with frequent patient visits are effective in attainment of normal motor and mental growth of patients with persistent CH.

Skordis et al (17) showed that the patients in the transient hypothyroidism group required lower doses of L-T4 for maintaining a normal thyroid hormone level. Similarly, Hashemipour et al (12) showed that higher doses of L-T4 are required in patients with persistent CH to attain normal TSH and fT4 levels. Indeed, also in our study, T4 doses(μg/kg/d) that maintained normal thyroid function tests were significantly higher at the end of first and second years in Group 1. Also, signs of thyrotoxicosis due to overtreatment were noted in 10 patients in the transient hyperthyrotropinemia group, while no such finding occurred in the CH group. In the transient hyperthyrotropinemia group, T4 dose requirement did not increase with weight gain and age, and TSH levels were maintained with lower T4 doses, a finding also in favor of transient hyperthyrotropinemia. To avoid unnecessary treatment and expenses as well as to decrease the risk of iatrogenic hyperthyroidism, we suggest that treatment may be discontinued in the first year or at the end of the second year and definitely before the third year in these infants.

In a study conducted by Gaudino et al (18), TSH levels were significantly higher following treatment discontinuation in patients with persistent hypothyroidism. In our study also, after one month of discontinuation of L-T4, TSH levels were significantly higher in Group 1 as compared to Group 2. Overall, serum TSH and fT4 levels should be remeasured latest at the end of the first month of life in patients with hyperthyrotropinemia. If treatment discontinuation is considered and the patient is left untreated, the mental outcome of the patients would be at risk. Increase in TSH levels following treatment discontinuation indicates that transient hyperthyrotropinemia was a misdiagnosis. T4 treatment should be reinitiated in such cases.

In conclusion, the results of the present study, conducted in a region where the frequency of hyperthyrotropinemia is noticeably high and where some of the patients are unnecessarily treated with L-T4 for long periods, show that none of the variables investigated in the study, with the exception of T4 doses needed to maintain a euthyroid state, could be used to distinguish between transient and permanent hyperthyrotropinemia (CH). For a definitive differential diagnosis between transient and permanent hyperthyrotropinemia, a reevaluation following a one month drug-free period at the end of 1 or 2 years of age is needed.

## Figures and Tables

**Table 1:**
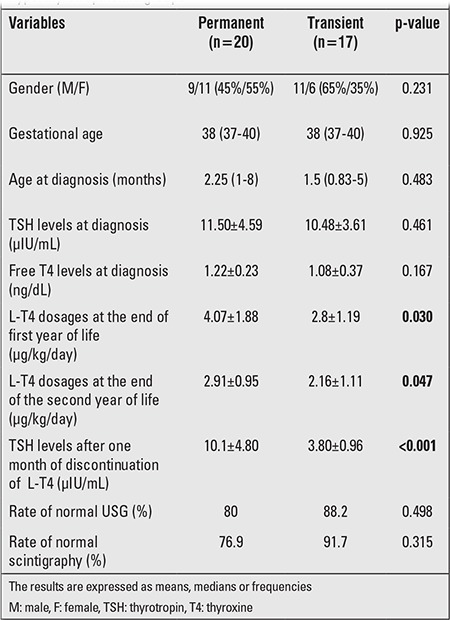
Comparison of clinical and laboratory characteristicsin the permanent congenital hypothyroidism and transienthyperthyrotropinemia groups
